# The Efficacy of Drug-Coated Balloon for Acute Coronary Syndrome

**DOI:** 10.1155/2023/4594818

**Published:** 2023-04-20

**Authors:** Hirokazu Naganawa, Akira Ito, Shinrou Saiki, Daisuke Nishi, Shinichi Takamatsu, Yoshihisa Ito, Takeshi Suzuki

**Affiliations:** Department of Cardiology, Toyokawa City Hospital, Toyokawa, Aichi, Japan

## Abstract

**Background:**

Percutaneous coronary intervention using a drug-eluting stent (DES) is a common therapeutic option for acute coronary syndrome (ACS). However, stent-associated complications, such as bleeding associated with dual antiplatelet therapy, in-stent restenosis, stent thrombosis, and neoatherosclerosis, remain. Drug-coated balloons (DCBs) are expected to reduce stent-associated complications. This study aimed to assess the efficacy of DCB therapy and compare it with that of DES therapy in patients with ACS.

**Materials and Methods:**

In this single-center, retrospective, observational study, we examined all patients with ACS treated with DCB or DES between July 2014 and November 2020. Patients with left main trunk lesions were excluded. The primary outcome was a composite of major adverse cardiovascular events (MACE: cardiac death, myocardial infarction, and target lesion revascularization) at one year.

**Results:**

Three hundred and seventy-two patients were treated with DES, and 83 patients were treated with DCB. MACE occurred in 10 (12.0%) patients in the DCB group and in 50 (13.4%) patients in the DES group (*P*=0.73).

**Conclusions:**

DCB is a valuable and effective therapy for patients with ACS. Moreover, DCB may become an alternative therapy for DES in patients with ACS.

## 1. Introduction

Acute coronary syndrome (ACS) is one of the leading causes of death worldwide [1–3]. Primary percutaneous coronary intervention (PCI) reportedly reduces cardiac events in patients with acute myocardial infarction (AMI) [4–7]. The use of second-generation drug-eluting stents (DES) is suggested and recommended in several clinical trials for safety and efficacy; therefore, PCI for ACS utilizing DES has recently become a common therapy [8–11]. However, stent-associated complications, such as bleeding event associated with dual antiplatelet therapy (DAPT), in-stent restenosis (ISR), stent thrombosis (ST), and neoatherosclerosis, remain. Drug-coated balloons (DCBs) are semicompliant angioplasty balloons covered with antirestenotic drugs, which are rapidly released locally into vessels during balloon inflation. Paclitaxel, which is coated on the balloon surface, is absorbed into the vessels and inhibits neointimal hyperplasia. Because the rationale behind DCB therapy is to combine balloon and drug therapy and does not involve leaving a permanent vascular implant, DCB is expected to reduce these stent-associated complications. DCB carries no risk of ISR or ST, and the native vessels maintain normal vascular function due to the absence of chronic inflammation caused by metallic struts and polymers. In addition, the duration of DAPT is reduced. Currently, DCB therapy is the standard treatment strategy for ISR [12–19] and small coronary vessel disease [20–22]. Although several studies have reported on the efficacy of DCB in ACS [23–25], there is still insufficient evidence. There is a paucity of current literature discussing the effects of DCB on the entire ACS population; therefore, this study aimed to assess the efficacy of DCB therapy and compare it with that of DES in patients with ACS.

## 2. Materials and Methods

This was a single-center, retrospective, observational study. We enrolled all patients with ACS who underwent PCI with DCB or DES at our hospital between July 2014 and November 2020. Patients with left main trunk lesions were excluded.

ACS was defined as a range of conditions compatible with acute myocardial ischemia and/or AMI, including ST-segment elevation myocardial infarction (STEMI), non-ST-segment elevation myocardial infarction (NSTEMI), and unstable angina pectoris (UAP) [26]. Diabetes mellitus was defined as hemoglobin A1c ≥6.5% or use of insulin or oral hypoglycemic drugs. Hypertension was defined as a systolic blood pressure (SBP) >140 mmHg, diastolic blood pressure >90 mmHg, or medical therapy for hypertension. Dyslipidemia was defined as total cholesterol ≥220 mg/dL, low-density lipoprotein cholesterol ≥140 mg/dL, high-density lipoprotein cholesterol ≤40 mg/dL, triglycerides ≥150 mg/dL, or the use of statins and/or lipid-lowering agents. A current smoker was defined as a smoker at the time of admission or one who had quit smoking within one year prior to admission. Left ventricular ejection fraction (LVEF) was measured using transthoracic echocardiography during the index hospitalization. LVEF was calculated using either the modified Simpson's method, Teichholz method, or eyeball estimation. The Teichholz method was adopted only when the modified Simpson method was unavailable. An eyeball estimate was adopted only when Simpson's and Teichholz's methods were unavailable. Cardiac shock was defined as SBP <90 mmHg, administration of vasopressors to maintain blood pressure, or attempted cardiopulmonary resuscitation. Bifurcation lesions were defined as lesions with a side branch ≥2.0 mm on visual assessment.

PCI was performed according to the standard technique. All procedures were performed through the radial or femoral artery using a 6–8 Fr guiding catheter. A bolus injection of heparin (8000 units) was administered after sheath insertion, and the activated clotting time (ACT) was maintained at >250 s with an additional bolus of heparin during the procedure [27]. All patients were pretreated with a loading dose of aspirin 300 mg, clopidogrel 300 mg, or prasugrel 20 mg before PCI according to the Japanese Circulation Society (JCS) guidelines [28]. The choice of either clopidogrel or prasugrel was at the discretion of the operator. All patients underwent intravascular ultrasound (IVUS) using either OptiCross (Boston Scientific, CA, USA) or AltaView (Terumo Medical, Tokyo, Japan). ACT was measured when PCI was completed and the sheath pulled. If ACT was >300 s, heparin was neutralized using protamine [29]. The following procedures were performed at the operator's discretion: lesion preparation and device selection for predilatation, thrombectomy and distal protection, final device selection, crossover therapy from DCB to DES, follow-up coronary angiography (CAG), duration of DAPT, and drugs administered for secondary prevention.

The operators used a paclitaxel-coated balloon (SeQuent Please, Nipro Corporation, Osaka, Japan) or a second-generation DES, considering the angiographical/IVUS findings and background of the patient. The inflation time of DCB had to be at least 30 s at optimal pressure. Whenever DCB was used, the operator carefully evaluated for further thrombus formation, acute recoil, and flow-limiting dissection in comparison with the results during the immediate phase and that after the 15-min phase. Bail-out stenting was carefully considered only in cases of residual stenosis of the lesion >50% (by visual estimation) after balloon dilatation with a sufficiently large balloon and/or dissection greater than or equal to type *C*, leading to acute vessel closure. Successful PCI was defined as a diameter stenosis <30% (by visual estimation) and thrombolysis in myocardial infarction (TIMI) flow grade ≥2 in the DCB group, and a diameter stenosis <20% and TIMI flow grade ≥2 in the DES group.

After the procedure, DAPT, a combination of aspirin (100 mg/day) and prasugrel (3.75 mg/day) or clopidogrel (75 mg/day) as a maintenance dose [28] was prescribed for 3–6 months for the DCB group or 6–12 months for the DES group, respectively. No routine follow-up CAG was performed. Follow-up CAG was performed if there were recurrent angina symptoms, silent ischemia detected by stress test and/or scintigraphy, or at the operator's discretion and planned at 6–8 months after the procedure for the DCB group or at 10–12 months for the DES group.

Quantitative coronary angiography (QCA) parameters were measured using a cardiovascular angiography analysis system (QAngio XA 7.3; Medis Medical Imaging Systems, Leiden, Netherlands). The values were obtained at three points: preprocedural PCI (baseline), postprocedural PCI (final), and follow-up CAG (follow-up). Lesion length, reference diameter (RD), minimal lumen diameter (MLD), and degree of stenosis (%DS) were measured, and late lumen loss (final MLD minus follow-up MLD) (LLL) was calculated. If the lesion was occluded, the MLD was 0 and %DS was 100, and the lesion length was calculated after thrombus aspiration or small balloon dilatation. Binary restenosis was defined as %DS >50% in the follow-up phase.

The primary outcome of this study was the occurrence of major adverse cardiovascular events (MACE), defined as cardiac death, myocardial infarction (MI), and target lesion revascularization (TLR) at one year. Cardiac death was defined as death resulting from cardiovascular causes such as AMI; sudden cardiac death, including unwitnessed death, heart failure, stroke, cardiovascular procedures, or cardiovascular hemorrhage; and death resulting from other cardiovascular causes [30]. MI was defined as an elevation of cardiac biomarker values above the upper limit in combination with at least one of the following: symptoms of myocardial ischemia, new ischemic electrocardiographic changes, development of new pathological *Q* waves, imaging evidence of new loss of viable myocardium or new regional wall motion abnormality, or identification of a coronary thrombus via angiography or autopsy [31]. TLR was defined as repeated PCI of the target lesion (including 10 mm proximal and 10 mm distal to the index device) or coronary artery bypass surgery on the target vessel due to evidence of ischemia, such as typical symptoms and/or positive laboratory testing [30]. The secondary outcome was the occurrence of MACE, ST, stroke, or bleeding. ST was defined according to the Academic Research Consortium criteria [32]. Bleeding was defined as Bleeding Academic Research Consortium (BARC) types 2, 3, and 5 [33].

All statistical analyses were performed using EZR (Saitama Medical Center, Jichi Medical University, Saitama, Japan), which is a graphical user interface for *R* (The *R* Foundation for Statistical Computing, Vienna, Austria) [34]. Continuous variables are expressed as means and standard deviations. For continuous data, groups of normally distributed and homoscedastic variables were compared using Student's *t*-test, while normally distributed and heteroscedastic variables were compared using the Welch test. Non-normally distributed variables were compared using the Mann–Whitney *U* test. Categorical variables are presented as numbers and percentages. For categorical data, groups were compared using the chi-squared test. Statistical significance was defined as a two-tailed *P* value <0.05.

All procedures were in accordance with the ethical standards of the institutional and/or national research committee and the 1964 Helsinki Declaration and its later amendments or comparable ethical standards. This study was approved by the Institutional Review Board of Toyokawa City Hospital. Informed consent was obtained from all the participants.

## 3. Results

During the study period, 83 and 372 patients were treated with DCB and DES, respectively. The baseline characteristics of the patients are shown in Table 1. The rates of prior PCI and MI were significantly higher in the DCB group than in the DES group. Clinical presentation differed between the DCB and DES groups (UAP was highest in the DCB group, while STEMI was highest in the DES group). Other baseline characteristics were not significantly different between the two groups. Medication after the procedure was similar in both groups, except for *β*-blockers.

The lesions and procedural characteristics are shown in Table 2. The target lesion and number of lesions were similar in both groups. De novo lesions were higher in the DES group, while bifurcation lesions were higher in the DCB group. Predilatation and the use of scoring/cutting balloons were higher in the DCB group. Inflation pressure was 9.4 and 11.9 atm (*P* < 0.01) and duration of inflation was 58.2 and 54.8 s (*P* < 0.01) in the DCB and DES groups, respectively.

QCA results are shown in Table 3. Lesion length and RD were significantly higher in the DES group. The %DS in the baseline phase was significantly lower in the DCB group; however, the %DS in the final phase was similar in both groups. Follow-up CAG was performed in 56 (67.5%) and 261 (70.2%) patients in the DCB and DES groups, respectively (*P*=0.63). LLL tended to be lower and restenosis was significantly higher in the DCB group.

Clinical outcomes are shown in Table 4. The primary outcome (MACE) occurred in 10 (12.0%) patients in the DCB group and 50 (13.4%) patients in the DES group (*P*=0.73). Cardiac death, MI, TLR, ST, and stroke were not significantly different between the groups. BARC 2, 3, 5 bleeding was significantly lower in the DCB group (0.0%) than in the DES group (4.6%) (*P*=0.04).

## 4. Discussion

The main findings of the present study are as follows: (1) the occurrence of MACE was not significantly different between the DCB and DES groups; and (2) BARC 2, 3, 5 bleeding was significantly lower in the DCB group than in the DES group.

DES therapy has improved clinical outcomes and prognosis and is considered the standard revascularization therapy for de novo lesions. However, DES therapy is associated with complications such as ISR, ST, neoatherosclerosis, and bleeding. With advancements in DCB therapy, stentless PCI has become an alternative to DES. Owing to the absence of chronic inflammation caused by metallic struts and polymers, DCB might have the advantages of early healing and preservation of normal vessel functions. DCB carries no risk of ISR or ST, and the duration of DAPT might be shortened. If the patient has a metal allergy, DCB could be safer than DES. In addition, an important consequence of DCB therapy is an increase in the lumen area after several months, known as late lumen enlargement (LLE). It is considered to be caused by a reduction of plaque volume, positive remodeling, repair of dissection, and some vessel healing mechanisms. Approximately 50.5–74% of patients show LLE after DCB therapy [35–38]. Onishi et al. reported that the American Heart Association (AHA)/American College of Cardiology (ACC) lesion type and rate of LLE were related [36]. In this study, 39.3% (22/56) of patients demonstrated LLE, with rates lower than those reported in previous studies. This was because the rate of AHA/ACC type B2 + C lesions in this study was 78.3%, which is higher than those reported in previous studies.

Bonaventura et al. reported that lesion preparation using a scoring balloon was associated with high procedural success and low target-lesion failure [39]. In this study, predilatation and the use of a scoring/cutting balloon were higher in the DCB group. PCI using DES can result in immediate luminal gain; however, stentless PCI using DCB cannot obtain a stent-like result. Therefore, lesion preparation resulting in sufficient luminal gain and controlled dissection is necessary to achieve good clinical outcomes. In this study, predilatation was performed in 100% of cases, and the usage rate of scoring/cutting balloons was 65.1% in the DCB group.

Furthermore, in this study, the rate of bifurcation lesions was higher in the DCB group than in the DES group. Generally, PCI for bifurcation lesions is associated with high procedural complications and worse outcomes [40]. Provisional stenting has been the gold standard for bifurcation lesions, and the two-stent strategy can be considered for narrowed large-side branches. However, the two-stent strategy has a higher risk of complications than the single-stent strategy. Therefore, an optimal strategy for the treatment of bifurcation lesions has not yet been established. However, Corballis et al. reported on the efficacy of the DCB strategy for bifurcation lesions [41]. DCB therapy could reduce the loss of side branches (owing to the absence of stent strut jailing) and shorten the procedure time. Many operators consider completing the procedure in a short time, particularly in cases of ACS; therefore, DCB therapy may be suitable for bifurcation lesions.

Wiviott et al. reported that the treatment for patients with ACS who were undergoing PCI and were administered prasugrel at a loading and maintenance doses of 60 mg and 10 mg, respectively, was associated with reduced ischemic events; however, it was associated with increased bleeding events compared with clopidogrel [42]. The European Society of Cardiology (ESC) and ACC/AHA/Society for Cardiovascular Angiography and Interventions (SCAI) guidelines recommend a loading and maintenance dose of prasugrel 60 mg and 10 mg or 5 mg, respectively [43, 44]. However, East Asians, including the Japanese, have a higher risk of bleeding than Western populations [45, 46] and have a different risk-benefit profile for antithrombotic therapy compared with Western populations [47]. Therefore, Saito et al. reported that a reduced dose of prasugrel (loading and maintenance doses of 20 mg and 3.75 mg, respectively) has confirmed the safety and efficacy in Japanese patients with ACS [48]. Accordingly, a reduced dose of prasugrel and a standard dose of prasugrel were recommended by the JCS guideline. In this study, we determined a loading and maintenance dose of prasugrel and clopidogrel according to the JCS guideline. In general, patients with ACS have a high risk of bleeding and ischemia [49]. Many previous studies have recommended that the optimal duration of DAPT be determined based on the risk of bleeding and ischemia [50–52]. In this study, the academic research consortium high bleeding risk (ARC-HBR) was 48.2% (40/83) and 41.9% (156/372) in the DCB and DES groups, respectively (*P*=0.30). The Japanese version of the HBR (J-HBR) [28] was 62.7% (52/83) and 66.9% (249/372) in the DCB and DES groups, respectively (*P*=0.46). Natsuaki et al. reported that the ARC-HBR and J-HBR rates were 48% and 64%, respectively [53], which are similar to our results. All bleeding events in this study occurred during DAPT therapy. In this study, we set the duration of DAPT at 3–6 months and 6–12 months for the DCB and DES groups, respectively. Because the duration of DAPT was longer in the DES group than in the DCB group, the rate of bleeding was higher in the DES group. Many clinical trials have been performed to shorten the duration of DAPT after DES implantation, including ACS [54–58], but the optimal duration of DAPT remains uncertain. Similarly, the optimal duration of DAPT for DCB therapy remains unclear; two trials reported a duration of 1–3 months [59, 60]. Theoretically, DCB could shorten DAPT due to the mechanism described above, and it has an advantage in HBR patients. A shorter DAPT duration with both DCB and DES may be possible in the future, and the superiority of DCB for bleeding risk may decrease.

Previous studies have evaluated the efficacy and feasibility of DCB therapy for STEMI [23], NSTEMI [24], and ACS [25]. Recently, Li et al. reported the efficacy and safety of DCB therapy in patients with AMI in a meta-analysis [61]. However, to the best of our knowledge, this is the first report involving only patients with ACS, including those with de novo and ISR lesions.

This study has several limitations. First, it was a nonrandomized, retrospective study; therefore, selection bias could not be excluded. Second, this study was performed at a single center with a small sample size. Third, the duration of DAPT in both groups was determined at the operator's discretion; therefore, it was not uniform. Fourth, owing to the small number of patients, we did not distinguish between in-hospital and postdischarge events. Finally, our follow-up data were limited to one year; longer follow-up data are necessary to evaluate the influence of rare clinical outcomes such as ST and neoatherosclerosis. Further prospective and randomized trials involving a large number of patients are necessary to confirm the results of this study.

## 5. Conclusions

DCB is an effective and valuable therapy for patients with ACS. Moreover, DCB therapy may become an alternative to DES in patients with ACS, especially in cases where bleeding complications are not desirable.

## Figures and Tables

**Table 1:** Baseline characteristics.

Characteristics	DCB	DES	*P* value
	(*n* = 83)	(*n* = 372)	
Age (years)	69.4 (±12.7)	68.4 (±12.1)	0.49

Male sex (*n* (%))	64 (77.1%)	293 (78.8%)	0.77

Weight (kg)	64.2 (±12.3)	64.5 (±13.9)	0.85

Body surface area (m^2^)	1.69 (±0.18)	1.69 (±0.21)	0.84

Body mass index	23.8 (±3.6)	24.1 (±3.8)	0.51

Diabetes mellitus (*n* (%))	25 (30.1%)	136 (36.6%)	0.34

Hypertension (*n* (%))	60 (72.3%)	244 (65.6%)	0.24

Dyslipidemia (*n* (%))	49 (59.0%)	227 (61.0%)	0.73

Current smoking (*n* (%))	20 (24.1%)	120 (32.2%)	0.15

Hemodialysis (*n* (%))	5 (6.0%)	10 (2.7%)	0.12

Atrial fibrillation (*n* (%))	8 (9.6%)	20 (5.4%)	0.14

Prior PCI (*n* (%))	33 (39.8%)	32 (8.6%)	<0.01

Prior MI (*n* (%))	25 (30.1%)	25 (6.7%)	<0.01

Prior CABG (*n* (%))	3 (3.6%)	5 (1.3%)	0.15

*Clinical presentation*			

STEMI (*n* (%))	21 (25.3%)	238 (64.0%)	<0.01

NSTEMI (*n* (%))	23 (27.7%)	59 (15.9%)	

UAP (*n* (%))	39 (47.0%)	75 (20.1%)	

LVEF (%)	54.3 (±12.9)	55.5 (±10.3)	0.43

*Medication*			

Aspirin (*n* (%))	81 (97.6%)	360 (96.8%)	0.70

Prasgurel (*n* (%))	74 (89.2%)	338 (90.9%)	0.63

Cropidogrel (*n* (%))	5 (6.0%)	23 (6.2%)	0.96

DOAC/warfarin (*n* (%))	6 (7.2%)	12 (3.2%)	0.09

ACE-I/ARB (*n* (%))	69 (83.1%)	318 (85.5%)	0.59

*β*-blocker (*n* (%))	53 (63.9%)	287 (77.2%)	0.01

Statin (*n* (%))	80 (96.4%)	341 (91.7%)	0.14

PPI (*n* (%))	80 (96.4%)	359 (96.5%)	0.96

*Note.* Continuous variables are expressed as mean and standard deviation. Categorical variables are presented as numbers and percentages. PCI: percutaneous coronary intervention; MI: myocardial infarction; CABG: coronary artery bypass graft; STEMI: ST-segment elevation myocardial infarction; NSTEMI: non-ST-segment elevation myocardial infarction; UAP: unstable angina pectoris; LVEF: left ventricular ejection fraction; ACE-I: angiotensin-converting enzyme-inhibitor; ARB: angiotensin receptor blocker; DOAC: direct oral anticoagulant; PPI: proton pump inhibitor.

**Table 2:** Lesion and procedural characteristics.

Characteristics	DCB	DES	*P* value
	(*n* = 83)	(*n* = 372)	
*Target vessels*			
LAD (*n* (%))	48 (57.8%)	180 (48.4%)	0.43
LCX (*n* (%))	18 (21.7%)	60 (16.1%)	
RCA (*n* (%))	17 (20.5%)	132 (35.5%)	

*Number of diseased vessels*			
1 (*n* (%))	48 (57.8%)	242 (65.1%)	0.26
2 (*n* (%))	29 (34.9%)	103 (27.7%)	
3 (*n* (%))	6 (7.3%)	27 (7.2%)	

De novo lesion (*n* (%))	58 (69.9%)	365 (98.1%)	<0.01

TRI (*n* (%))	65 (78.3%)	259 (69.6%)	0.11

Thrombus burden (*n* (%))	62 (74.7%)	308 (82.8%)	0.09

Calcified lesion (*n* (%))	8 (9.6%)	63 (16.9%)	0.10

AHA/ACC type B2 + C lesion (*n* (%))	65 (78.3%)	281 (75.5%)	0.59

Bifurcation lesion (*n* (%))	24 (28.9%)	63 (16.9%)	0.01

Distal protection (*n* (%))	38 (45.8%)	209 (56.2%)	0.09

Thrombectomy (*n* (%))	18 (21.7%)	117 (31.4%)	0.08

Bailed out stenting (*n* (%))	2 (2.4%)	—	—

Cardiac shock (*n* (%))	1 (1.2%)	20 (5.4%)	0.10

IABP (*n* (%))	4 (4.8%)	40 (10.8%)	0.10

V-A ECMO (*n* (%))	1 (1.2%)	5 (1.3%)	0.91

*Preprocedural TIMI flow grade*			
1 (*n* (%))	18 (21.7%)	177 (47.6%)	<0.01
2 (*n* (%))	6 (7.2%)	22 (5.9%)	
3 (*n* (%))	21 (25.3%)	66 (17.7%)	
4 (*n* (%))	38 (45.8%)	107 (28.8%)	

*Postprocedural TIMI flow grade*			
1 (*n* (%))	0 (0.0%)	0 (0.0%)	0.11
2 (*n* (%))	0 (0.0%)	0 (0.0%)	
3 (*n* (%))	0 (0.0%)	11 (3.0%)	
4 (*n* (%))	83 (100.0%)	361 (97.0%)	

Pre-dilatation (*n* (%))	83 (100.0%)	230 (61.8%)	<0.01

Scoring/cutting balloon use for lesion preparation (*n* (%))	54 (65.1%)	41 (11.0%)	<0.01

Postdilatation (*n* (%))	1 (1.2%)	95 (25.5%)	<0.01

Devices diameter (mm)	2.66 (±0.41)	3.15 (±0.52)	<0.01

Devices length (mm)	20.3 (±3.8)	22.6 (±7.7)	<0.01

Inflation pressure (atm)	9.4 (±3.0)	11.9 (±1.5)	<0.01

Duration of inflation (s)	58.2 (±12.7)	54.8 (±11.0)	<0.01

*Note.* Continuous variables are expressed as mean and standard deviation. Categorical variables are presented as numbers and percentages. LAD: left anterior descending artery; LCX: left circumflex; RCA: right coronary artery; TRI: transradial intervention; AHA: American Heart Association; ACC: American College of Cardiology; IABP: intraaortic balloon pumping; V-A ECMO: veno-arterial extracorporeal membrane oxygenation; TIMI: thrombolysis in myocardial infarction.

**Table 3:** Initial and follow up quantitative angiographic results.

Initial QCA	DCB	DES	*P* value
	(*n* = 83)	(*n* = 372)	

Lesion length (mm)	14.0 (±8.1)	19.1 (±11.8)	<0.01
Reference diameter (mm)	2.35 (±0.60)	2.61 (±0.64)	<0.01
*Minimal lumen diameter (mm)*			
Baseline	0.63 (±0.42)	0.45 (±0.45)	<0.01
Final	1.87 (±0.47)	2.49 (±0.54)	<0.01
*Degree of stenosis (%)*			
Baseline	72.0 (±17.5)	82.5 (±18.1)	<0.01
Final	19.1 (±10.3)	16.9 (±9.9)	0.09

Follow up QCA	DCB	DES	
	(*n* = 56)	(*n* = 261)	

Reference diameter (mm)	2.42 (±0.49)	2.70 (±0.57)	<0.01
Minimal lumen diameter (mm)	1.72 (±0.71)	2.13 (±0.69)	<0.01
Degree of stenosis (%)	29.3 (±24.4)	23.1 (±18.4)	0.11
Late lumen loss (mm)	0.14 (±0.67)	0.34 (±0.68)	0.07
Angiographic restenosis (*n* (%))	10 (17.9%)	19 (7.3%)	0.01

*Note.* Continuous variables are expressed as mean and standard deviation. Categorical variables are presented as numbers and percentages. QCA: quantitative angiographic results.

**Table 4:** Clinical outcomes at one year.

Characteristics	DCB	DES	*P* value
	(*n* = 83)	(*n* = 372)	
MACE	10 (12.0%)	50 (13.4%)	0.73
Cardiac death	2 (2.4%)	20 (5.4%)	0.25
MI	0 (0.0%)	9 (2.4%)	0.15
TLR	8 (9.6%)	21 (5.6%)	0.18
Stent thrombosis	0 (0.0%)	3 (0.8%)	0.41
Stroke	1 (1.2%)	3 (0.8%)	0.73
BARC 2, 3, 5 bleeding	0 (0.0%)	17 (4.6%)	0.04

*Note.* Categorical variables are presented as numbers and percentages. MACE: major adverse cardiovascular events; MI: myocardial infarction; TLR: target lesion revascularization; BARC: Bleeding Academic Research Consortium.

## Data Availability

The datasets generated during and/or analyzed during the current study are available from the corresponding author on reasonable request.
